# Rapid screening of cellular stress responses in recombinant *Pichia pastoris* strains using metabolite profiling

**DOI:** 10.1007/s10295-017-1904-5

**Published:** 2017-02-03

**Authors:** Gregory D. Tredwell, Rochelle Aw, Bryn Edwards-Jones, David J. Leak, Jacob G. Bundy

**Affiliations:** 1 Department of Surgery and Cancer, Faculty of Medicine, Imperial College London, London, SW7 2AZ UK; 2 Department of Applied Mathematics, Australian National University, Canberra, Australia; 3 Department of Life Sciences, Faculty of Natural Sciences, Imperial College London, London, SW7 2AZ UK; 4 FUJIFILM Diosynth Biotechnologies, Billingham, TS23 1LH UK; 5 Department of Biology and Biochemistry, University of Bath, Bath, BA2 7AY UK

**Keywords:** Metabolic footprinting, Unfolded protein response, Clonal variation, Bioprocessing, Recombinant protein

## Abstract

Heterologous protein production in the yeast *Pichia pastoris* can be limited by biological responses to high expression levels; the unfolded protein response (UPR) is a key determinant of the success of protein production in this organism. Here, we used untargeted NMR metabolic profiling (metabolomics) of a number of different recombinant strains, carried out in a miniaturized format suitable for screening-level experiments. We identified a number of metabolites (from both cell extracts and supernatants) which correlated well with UPR-relevant gene transcripts, and so could be potential biomarkers for future high-throughput screening of large numbers of *P. pastoris* clones.

**Electronic supplementary material:**

The online version of this article (doi:10.1007/s10295-017-1904-5) contains supplementary material, which is available to authorized users.

## Introduction

The yeast *Pichia pastoris* is an important platform for process-scale production of recombinant secreted proteins. It can be grown to very high cell densities in bioreactors, under forced aeration, to improve yields. However, the specific productivity is often low. Simply increasing promoter strength for recombinant genes is not a panacea, as biological feedback loops may result in decreased levels of secreted protein. A key mechanism in this regard is a homeostatic response to the buildup of unfolded protein in the endoplasmic reticulum (ER), the unfolded protein response (UPR), a transcriptional programme that is regulated by activation at UPR elements (UPRE) by a transcription factor generated by splicing of *HAC1* mRNA [21]. Although there is some argument about the detailed mechanism, in one model an increase of unfolded protein in the ER binds the chaperone Kar2p, causing it to dissociate from the transmembrane protein Ire1p. This then activates a cytoplasmic endoribonuclease activity in Ire1p, allowing it to splice *HAC1* mRNA [11], generating a transcriptionally competent and active gene encoding the transcription factor, Hac1p. This binds to UPRE sites in UPR-responsive promoter regions, including *HAC1*, enhancing the expression. As many of the UPR-responsive genes encode chaperones and related factors which can enhance protein folding, this leads to a positive feedback loop.

Low levels of UPR can improve protein production by increasing the expression of chaperones, but high levels of UPR can decrease it by shutting down translation altogether and activating the ER-associated degradation (ERAD) pathway leading to protein degradation [7, 25]. As the UPR is so critical for protein secretion, it is often used as an indicator to determine whether cells are stressed, and based on the premise that stressed cells will result in lower secretion, this is often used in strain design to find good secretors. However, traditional methods for screening for UPR require separate steps for extracting RNA and then determining transcript levels, or analysis of protein concentrations, which can also be time consuming and laborious. Therefore, any biological indicator that could be used to estimate potential levels of cellular stress could be very valuable.

A key step in scaling up secreted protein production is in selection of strains for testing at larger volumes. There is a wide variation in secretion levels in recombinant clones, and companies often screen thousands of colonies to identify the best secretor before scaling up to the bioreactor culture [6, 27]. Metabolic profiling is widely used as a strategy to report on cellular physiology. Metabolite changes within a cell are extremely dynamic, so metabolic profiles give a real-time picture of cellular responses and metabolites form arguably the most direct representation of cellular phenotype [23]. Furthermore, metabolomic results are readily converted into target assays well suited to rapid screening and/or online monitoring [5]. There is also considerable interest in using metabolic profiling of bioprocess supernatants for rational optimization of medium composition [2, 16, 24].

Metabolomic analysis has been used to determine the impact of recombinant protein expression in *P. pastoris* under varying growth conditions, including oxygen limitation and glucose–methanol co-utilization [4, 14]. Furthermore, Jorda et al. used the combination of metabolic flux analysis and metabolomic analysis to determine the impact on metabolism of expressing *Rhizopus oryzae* lipase: trehalose levels were significantly upregulated, indicating an increased flux through the ATP futile cycle, which may be related to the induction of the UPR [15]. These investigations into the use of metabolomics analysis provide a basis for potential metabolic engineering that may assist with identifying bottlenecks in the production of recombinant proteins. Here, we generated metabolic profiles for a series of *P. pastoris* constructs that varied in their levels of UPR induction and carried out a proof-of-principle study to determine if there were any potential metabolite biomarkers of UPR.

## Materials and methods

### Strains and growth conditions

*Pichia pastoris* GS115 (recently reclassified as *Komagataella* sp. [17]) was obtained from Invitrogen, Paisley, UK. The three-copy trypsinogen strain was generated as previously described [10]. The human lysozyme strains were a gift from Prof. David Archer (Nottingham) [28]. The Mfe-23 and BC1 strains were a gift from Dr. Kate Royle. *P. pastoris* strains were grown in 24-well plates (Corning, NY, USA) in BMG [100 mM potassium phosphate, pH 6.0, 1.34% (w/v) yeast nitrogen base (YNB), 4 × 10^–5^% (w/v) D-Biotin, 1% (v/v) glycerol)] for 24 h. Cells were centrifuged for 5 min at 4000 rpm at room temperature and the supernatant removed. The cultures were then re-suspended in the methanol-containing medium BMM for expression (as BMG but with 0.5% v/v methanol replacing glycerol) and incubated for 4 h.

#### Reverse transcription-qPCR

For reverse transcription (RT)-qPCR, RNA was isolated using RiboPure Yeast Kit, according to the manufacturer’s instructions (Applied Biosystems, Warrington, UK). cDNA was prepared using the High-Capacity cDNA Archive Kit (Applied Biosystems, Warrington, UK). 1 μg RNA was used in a total reaction volume of 20 μL. RT-qPCR reactions were set up using the 2X SYBR^®^ Green JumpStart Taq Ready Mix (Sigma-Aldrich, Dorset, UK). A Chromo4™ Real-Time Detector using the thermal cycler software Opticon 3 (Bio-Rad, Hemel Hempstead, UK) was used. Data were analysed using the Pfaffl method, based on ΔΔ-Ct [19, 22] and normalized to ACT1 as the housekeeping gene. Primers for ACT1 were GCT TTG TTC CAC CCA TCT GT and TGC ATA CGC TCA GCA ATA CC. Primers for HAC1 were CGA CTA CAT TAC TAC AGC TCC ATC A and TGC TGT AAT GTG TAA AGA TGA ATC C, for PDI, GCC GTT AAA TTC GGT AAG CA and TCA GCT CGG TCA CAT CTT TG and for KAR2, TCA AAG ACG CTG GTG TCA AG and TAT GCG ACA GCT TCA TCT GG.

##### Metabolite analysis

Extracellular and intracellular metabolite concentrations were measured by ^1^H NMR. For extracellular metabolite measurements, samples from the incubation plate (1 ml) were quickly centrifuged and decanted before being frozen on dry ice. For intracellular metabolite measurements, a total quenching procedure of whole broth (cells + media) with 60% aqueous methanol and 0.11 M ammonium bicarbonate was used to halt metabolism, as previously described [26]. The samples were dried under reduced pressure and reconstituted in 600 μl of NMR buffer (1 mM trimethylsilyl-2,2,3,3-tetradeuteropropionic acid (TSP), 0.1 M phosphate buffer, pH 7.4, in 95% D_2_O). Samples (550 μl) were transferred to 5 mm NMR tubes and spectra were acquired on a Bruker Avance DRX600 NMR spectrometer (Bruker BioSpin, Rheinstetten, Germany), equipped with a 5 mm inverse probe, operating at a ^1^H frequency of 600 MHz. Samples were introduced with an automatic sampler and spectra were acquired using standard approaches (Dona, 2014). Briefly, 2D JRES ^1^H NMR spectra were acquired with the pulse-sequence d1-90y-τ-180-τ-acquire FID, with suppression of the water resonance during d1. Data were acquired into 32 k data points in F2 with a d1 of 2 s and 16 transients using 32 increments of τ; the spectral widths in F2 and F1 were 12,019 and 50 Hz, respectively. Spectra were processed in iNMR 3.4 (Nucleomatica, Molfetta, Italy). Fourier transform of the free-induction decay was applied with a line broadening of 0.5 Hz. Metabolite peak integrals were obtained with rNMR [18] and expressed relative to TSP (Supplementary Material).

## Results and discussion

We used eight different strains expressing different recombinant proteins (Table 1) and grew them in miniaturized cultures in 24 deep-well microtitre plates. Strains with either one or three copies of the trypsinogen gene and human lysozyme (HuL) mutational variants that have been shown to have different stabilities were used, as these have been shown to induce the UPR [13, 28]. Hesketh et al. [12] selected two of the mutant variants, T70N and I56T, and using continuous culture observed variations using RNAseq. Although they determined “reporter metabolites” for these two strains, this was done solely through annotations of the transcriptomic data and did not include any direct metabolite analysis. We also included BC1 and Mfe-23, two single chain antibody fragments (ScFvs), which were chosen as these are industrially relevant products, showing the applicability of our work in an industrial context. All genes were expressed using the *AOX1* promoter and α-mating factor (α-MF) secretion signal from *Saccharomyces cerevisiae* that targets the protein to the secretory pathway. Cultures were grown for 24 h in a buffered glycerol minimal medium and then switched to a buffered methanol minimal medium to induce protein expression. Samples were taken after 4 h of induction. We monitored UPR levels by measuring *HAC1*, *KAR2* and *PDI1* transcripts by qRT-PCR using *ACT1* as a reference gene. All strains were normalized to wild type to allow for the background of potential cellular stress effects caused by the switch to methanol medium [10]. The two trypsinogen strains had the highest levels of *HAC1* induction.

**Table 1:** Summary table of strains used in this study indicating the recombinant protein expressed, dry cell weight (g/L) growth after 24 h in glycerol, followed by 4 h expression in methanol-containing medium and fold difference of transcript levels of *HAC1*, *KAR2* and *PDI1* compared to wild-type GS115

Strain	Recombinant protein	Source	Dry cell weight (g/L)	Fold difference compared to GS115 at 0 h		
				*HAC1*	*KAR2*	*PDI1*
T1	Trypsinogen (1 copy)	[15]	0.492 ± 0.175	2.132 ± 0.656	−0.353 ± 0.603	0.511 ± 0.326
T3	Trypsinogen (3 copy)	[15]	0.436 ± 0.072	2.912 ± 0.314	0.392 ± 0.753	1.378 ± 0.826
L	Lysozyme (wild-type)	[20]	0.990 ± 0.592	0.307 ± 0.412	0.145 ± 0.225	0.170 ± 0.527
I8	Lysozyme (I89V variant)	[20]	1.128 ± 0.439	−0.268 ± 1.117	0.073 ± 0.446	0.491 ± 0.591
T7	Lysozyme (T70A variant)	[20]	0.895 ± 0.489	0.608 ± 0.600	0.200 ± 0.563	0.532 ± 0.475
I5	Lysozyme (I56T variant)	[20]	0.866 ± 0.173	−0.327 ± 0.675	0.567 ± 0.333	0.504 ± 0.340
B	BC1	Unpublished work in our laboratory	0.563 ± 0.058	1.173 ± 0.090	−1.130 ± 0.912	0.246 ± 0.606
M	Mfe23	Unpublished work in our laboratory	0.651 ± 0.172	0.843 ± 0.440	−1.295 ± 1.206	0.487 ± 0.540

All data represented as the average of three biological repeats with standard deviation shown

We carried out untargeted proton NMR metabolic profiling of both the exometabolome (i.e. cell supernatants) and “total quenching” of both exo- and endometabolome (i.e. cells + supernatants together). Total quenching of *P. pastoris* broths combined with NMR profiling gives useful data on intracellular metabolites that contain effectively as much information as does quenching in cold solvent and removing the supernatant [26]. Most metabolites showed quite different patterns between the exometabolome and the total quench results, with the exception of some organic acids (formate, 2-oxoisocaproate, 3-methyl-2-oxovalerate), which we already knew to be supernatant contaminants, and glycerophosphocholine; the implication is that the culture supernatant and the total quench (whole broth) data represent two independent datasets, both of which could contain potential biomarkers.

**Fig. 1:**
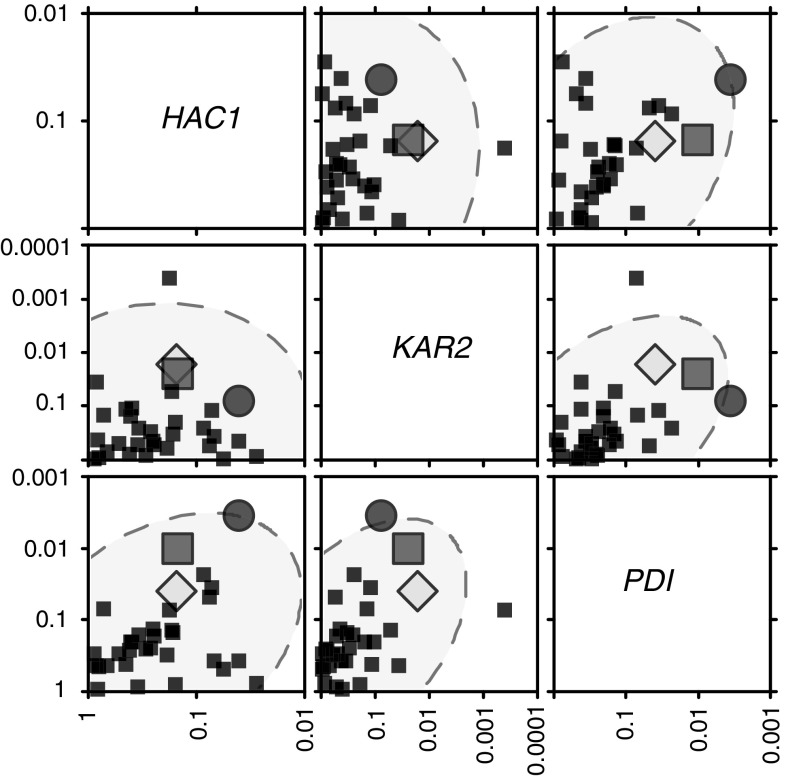
Metabolites associated with genes relevant to the unfolded protein response; axes represent *P* values derived from partial correlations. Metabolites were measured by 2D J-resolved NMR spectroscopy using standard approaches [8], which offers benefits of reduced overlap between resonances in the crowded ^1^H spectrum, and individual metabolite peaks were selected and quantitated using rNMR [15]. Each dot represents a single metabolite; *blue dot* isoleucine; *red square* aspartate; *yellow diamond* arabitol (color figure online)

There were some robust correlations between the individual gene expression levels (*HAC1*, *KAR2*, *PDI1*) and individual metabolites, although there were no metabolites which were significantly correlated for all three genes (Fig. 1). Induction of *HAC1* under these conditions can be used as an indicator of UPR, as this is assumed to reflect activation at the UPRE in the promoter region by Hac1p. The lack of complete correlation is only to be expected, as there were no strong correlations between the different transcript levels: *HAC1* vs. *PDI1* was the only pair that was significantly related at the 0.05 level (*P* = 0.024), but the relationship was still weak (*r* ^2^ = 0.21). However, *HAC1* expression was also strongly associated with the extent of growth for each strain (as represented by dry cell weight, DCW). Because of this, we also calculated partial correlations for each gene in turn against both DCW and metabolite concentration, i.e. representing the gene/metabolite association once the effect of DCW has been allowed for.

In general, *HAC1* levels were much more strongly associated with DCW than individual metabolite concentrations based on total quenching, whereas the relative effect of DCW was much less for *KAR2* and *PDI1* (Fig. S1). There were no metabolites which were associated with all three genes at the *P* < 0.05 level, but on comparing across all three, it was possible to identify some potential biomarker candidates: arabitol, aspartate and isoleucine were among the most highly associated metabolites for all genes (Fig. 1). (The associations were much weaker for the supernatant metabolites (Fig. S2), so only the total quenching results are discussed from here on.)

Why were those specific metabolites correlated with gene expression? Arabitol is produced as an osmolyte by many fungi, including *P. pastoris*. It probably acts here as part of a general mechanism to reduce cell stress: different stresses in *P. pastoris*, including misfolded protein, can increase arabitol levels [1, 9]. For aspartate and isoleucine, though, it is also worth testing whether the changes in intracellular amino acid pool sizes were associated with the amino acid requirement of the different recombinant proteins—i.e. were Asp and Ile particular bottlenecks for the recombinant proteins? There are some severe limitations to this: for instance, the data we have on the amino acid composition of *P. pastoris* do not distinguish between Gln and Glu, or Asp and Asn, and so we had to combine data for Glx and Asx [3]; and the recombinant protein composition data are obviously not different for the one-copy and three-copy trypsinogen strain and differ only slightly for the lysozyme strains. Nonetheless, as the mismatch for Asp and Ile fall within the range of all the amino acids for all the strains (Fig. S3), we conclude that there is no evidence of an association with amino acid requirements. Similarly, Carnicer et al. also found no association between amino acid pools and recombinant protein sequence for *P. pastoris*, although this was for the different case of a single protein grown under multiple conditions [4]. Additionally, Jorda et al. found the same result when comparing an expressing and non-expressing strain of Rol. However, it was determined that an adaptation of the amino acid concentration distribution may have been as a consequence of additional energy demands during recombinant protein production (with particular reference to an increase in trehalose) [14, 15].

## Conclusions

Our current data show that monitoring UPR levels by gene transcription is certainly feasible at a potentially high-throughput miniaturized scale and can therefore address a key bottleneck in the pathway to produced secreted proteins. However, this is relatively time consuming and laborious to do by traditional amplification-based methods, once all the different steps required have been allowed for [20]. We have found robust correlations between metabolites and *KAR2*/*PDI1* transcript levels, in particular (once DCW has been allowed for), which indicate that metabolites could potentially be used as proxy indicators of UPR stress. Metabolite biomarkers have high precision and are well suited to high-throughput sampling and analysis, and therefore offer an alternative approach to screen for clones with low levels of UPR. Furthermore, this approach could be easily extended to process monitoring in bioreactors, especially as some of the potential biomarkers included supernatant metabolites.

## Electronic supplementary material

Below is the link to the electronic supplementary material.

Supplementary material 1 (PDF 109 kb)

Supplementary material 2 (XLSX 69 kb)
