# Supplementary material for: Rapid screening of cellular stress responses in recombinant Pichia pastoris strains using metabolite profiling
Source: J Ind Microbiol Biotechnol. 2017 Feb 3;44(3):413–7. doi: 10.1007/s10295-017-1904-5 (PMC5329079; doi:10.1007/s10295-017-1904-5)
Supplement: Supplementary file 1 — Supplementary material 1 (PDF 109 kb) [file 10295_2017_1904_MOESM1_ESM.pdf]

Supplementary figures

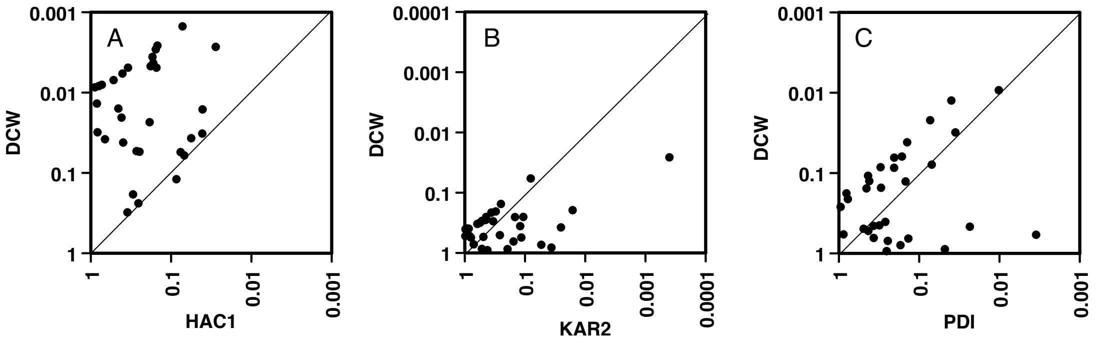

Figure S1. Comparison of relative importance of DCW or gene expression as calculated by partial correlation across all metabolites. Points falling in the upper left half (above the line x=y) represent metabolites more influenced by DCW than gene expression, and points falling in the lower right half are more influenced by gene expression than DCW. A: *HAC1*; B: *KAR2*; C: *PDI1*.

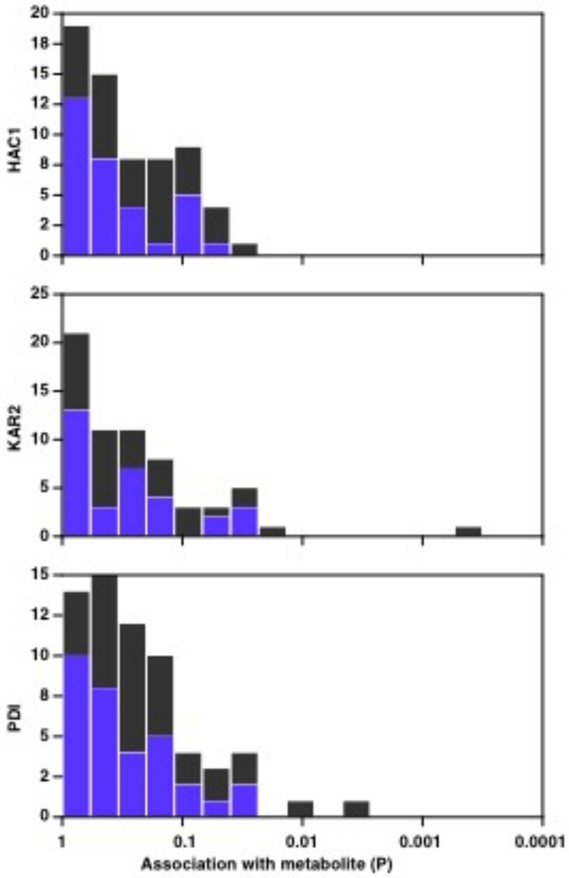

Figure S2. Associations between gene expression and metabolite levels are higher for extracted (black) than supernatant (blue) metabolites.

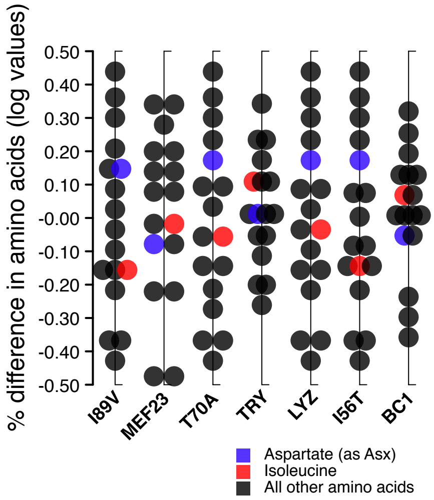

Figure S3. Aspartate (blue dots, as Asx) and isoleucine (red dots) are not unusually discongruent to the amino acid composition of *Pichia pastoris* when compared across all amino acids. (Glu and Gln represented as Glx; Trp was the most outlying amino acid for all recombinant proteins, and is omitted here for clearer plotting.)
